# Investigating the Retained Inhibitory Effect of Cobimetinib against p.P124L Mutated MEK1: A Combined Liquid Biopsy and in Silico Approach

**DOI:** 10.3390/cancers14174153

**Published:** 2022-08-27

**Authors:** Cristina Catoni, Cristina Poggiana, Antonella Facchinetti, Jacopo Pigozzo, Luisa Piccin, Vanna Chiarion-Sileni, Antonio Rosato, Giovanni Minervini, Maria Chiara Scaini

**Affiliations:** 1 Immunology and Molecular Oncology Unit, Veneto Institute of Oncology, IOV-IRCCS, 35128 Padua, Italy; 2 Department of Surgery, Oncology and Gastroenterology, Oncology and Immunology Section, University of Padua, 35128 Padua, Italy; 3 Melanoma Oncology Unit, Veneto Institute of Oncology, IOV-IRCCS, 35128 Padova, Italy; 4 Department of Biomedical Sciences, University of Padua, 35121 Padua, Italy

**Keywords:** liquid biopsy, circulating melanoma cells, circulating tumor DNA, BRAF/MEK inhibitors, drug resistance, molecular dynamics simulation

## Abstract

**Simple Summary:**

This work focuses on the peculiar contribution made by molecular dynamics simulation and in silico tools, in the choice of an effective second line therapy for a BRAF-mutated melanoma patient who developed resistance to the undergoing targeted therapy with BRAF/MEK inhibitors. Among the MEK inhibitors, we identified a drug alternative to trametinib, able to block the target even in the presence of a damaging mutation, and supported these findings, gathered by an in silico approach, with a liquid biopsy tracking of the response to treatment. The evolution of the disease, before and after the therapy change, was followed by analysis of the circulating tumor DNA and circulating melanoma cells.

**Abstract:**

The systemic treatment of metastatic melanoma has radically changed, due to an improvement in the understanding of its genetic landscape and the advent of targeted therapy. However, the response to BRAF/MEK inhibitors is transitory, and big efforts were made to identify the mechanisms underlying the resistance. We exploited a combined approach, encompassing liquid biopsy analysis and molecular dynamics simulation, for tracking tumor evolution, and in parallel defining the best treatment option. The samples at different time points were collected from a BRAF-mutant melanoma patient who developed an early resistance to dabrafenib/trametinib. The analysis of the circulating tumor DNA (ctDNA) identified the MEK1 p.P124L mutation that confers resistance to trametinib. With an in silico modeling, we identified cobimetinib as an alternative MEK inhibitor, and consequently suggested a therapy switch to vemurafenib/cobimetinib. The patient response was followed by ctDNA tracking and circulating melanoma cell (CMC) count. The cobimetinib administration led to an important reduction in the BRAF p.V600E and MEK1 p.P124L allele fractions and in the CMC number, features suggestive of a putative response. In summary, this study emphasizes the usefulness of a liquid biopsy-based approach combined with in silico simulation, to track real-time tumor evolution while assessing the best treatment option.

## 1. Introduction

In the era of precision medicine, the role and impact of targeted drugs have reached a position of unquestionable importance. Consequently, accurate computational approaches have proved useful resources in the process of drug development [1]. Moreover, with the improvement of computational methods, the designing, modeling, binding energy and kinetics’ predictions, have become much more feasible [1]. At the same time, similar approaches have also gained much more attention in the effort of overcoming tumor resistance by assessing the interaction of specific drugs with targets that gained a specific mutation as an escape strategy [2]. Indeed, resistance to treatment is a phenomenon still difficult to predict and/or prevent, and represents the major obstacle to the long-term effectiveness of cancer therapy [3]. 

Since an impressive mass of experimental results on resistance mechanisms was obtained, and high-throughput data were gathered, in silico modeling and computational predictions have become increasingly important, as they can putatively provide companion insights about resistance mechanisms, yielding suggestions about promising treatment alternatives [4]. 

Cutaneous malignant melanoma is the most lethal form of skin cancer and can be considered as a paradigm of an exceptionally aggressive, highly heterogeneous and complex disease [5,6,7]. These characteristics make the management of metastatic melanoma patients, and their relapse, particularly difficult and full of hindrances. Targeted drugs and immune checkpoint inhibitors are the standard therapy in patients with metastatic melanoma. 

A combination of a BRAF and a MEK inhibitor (BRAFi/MEKi-combined targeted therapy) has revolutionized the management of metastatic melanoma patients harboring a BRAF mutation at codon 600, significantly improving their overall survival (OS) and progression-free survival (PFS) [8]. Three BRAFi/MEKi combinations are currently approved by the FDA for the treatment of metastatic melanoma, including dabrafenib plus trametinib, vemurafenib plus cobimetinib and encorafenib plus binimetinib [9]. Despite the fact that the BRAFi/MEKi-combined targeted therapy is more efficacious in comparison to the traditional therapies (chemotherapies, monotherapies), about 20% of patients do not benefit from the treatment due to pre-existing genetic alterations (intrinsic or primary resistance), and about 50% of patients relapse within 12 months from the beginning of the treatment due to the emergence of mutations that reactivate the MAPK pathway or activate alternative pathways to support the tumor growth (acquired resistance) [10,11,12]. Hence, the early identification of primary or acquired resistance mechanisms should become a priority in order to select the most effective treatment option at the very outset. Due to the heterogeneous and polyclonal nature of the metastatic disease, the analysis of a single biopsy may not represent the whole tumor genetic landscape, with important consequences for the tracking of disease evolution [13]. Liquid biopsy has emerged as a non-invasive tool encompassing biomarkers with high translational potential, due to their ability to provide comparable, or even more detailed, information than conventional tissue biopsy [14,15]. Liquid biopsy can be representative of a sum of different tumor clones, both those that are the most aggressive and drug-resistant (mostly contributing to the vital component of the circulating tumor cells), and those that are responsive to the therapy (mostly concurring to the apoptotic circulating tumor-cell fraction), and their molecular characteristics are also represented, in varying proportions, by the circulating tumor DNA (ctDNA) [16,17,18].

Moreover, the analysis of the circulating melanoma cells (CMCs) and ctDNA has proved to be a useful tool for improving risk assessment, the real-time monitoring of therapeutic efficacy, as well as the early detection of recurrence, and monitoring of tumor evolution with a minimally invasive blood draw [19,20,21]. 

In the current study, we report on the success of a combined approach based on liquid biopsy tracking plus molecular dynamics simulation, in following the real-time evolution of the disease in a stage IV melanoma patient. The effect of therapy change, guided by the in silico analysis assessment, was followed longitudinally to detect the suggestive signs of response.

## 2. Materials and Methods

### 2.1. Patient and Samples

The patient was enrolled at the time of metastatic disease diagnosis, within a pilot study for stage IV cutaneous melanoma patients at the Veneto Institute of Oncology, Italy (2019–2023). The study was approved by the local Ethics Committee, conducted according to the Declaration of Helsinki, and the patient provided written informed consent to be part of the study. The patient commenced with dabrafenib/trametinib treatment, and was followed with serial blood sampling to monitor the disease evolution. The peripheral blood was collected at baseline, before starting the therapy (T0), at the time of progression (T1), at the time of the shift to a new line of treatment (T2) and one month after the start of the new therapy (T3). The blood samples were collected into a 10 mL tube containing CellSave Preservative (Menarini Silicon Biosystems, Bologna, Italy) for the CMC count and in Streck Cell-Free DNA BCT tubes (Streck, La Vista, NE, USA) for the circulating cell-free DNA (cfDNA) analysis. The blood plasma was obtained by double centrifugation and stored at −80 °C until tested by next-generation sequencing (NGS) and droplet digital PCR (ddPCR). The samples for CMC enumeration were processed within 96 hours from the blood draw.

### 2.2. Molecular Dynamics Simulation

The crystal structure of MEK1 in complex with the allosteric MEK inhibitor, cobimetinib (PDB code: 4LMN), was used as a starting point for the simulations. The simulation environment was prepared using the CHARMM-GUI Input Generator [22], with calculations carried out with GROMACS [23] using the CHARMM36m force field and the TIP3p explicit solvent model. The parameters for cobimetinib were calculated using the OpenFF tool integrated in the CHARMM-GUI server. The simulation run protocol consisted of 100 conjugate gradient minimization steps, 1000 ps under NVT conditions followed by 500 ns of classic molecular dynamics simulation at 310 K and 1.01325 bar. The simulations were performed in triplicate and the resulting trajectories were compared in terms of root mean square deviation (RMSD) and root mean square fluctuation (RMSF). The RING 3.0 server [24] was used to estimate the variation in residue–residue interaction network around the mutated amino acid position.

### 2.3. cfDNA Analysis

A hybridization capture-based target enrichment custom panel (SureSelect Cancer All-In-One custom panel; Agilent Technologies, Santa Clara, CA, USA) was used for the detection of the single nucleotide variants (SNVs) and the small deletions/insertions of 52 genes, plus copy number variations (CNV) for 11 genes (Table S1, Supplementary Materials). The design covered hotspots for the driver and targetable mutations, together with the genes involved in the pathways associated with resistance to treatment and/or disease outcome both for cutaneous and uveal melanoma [25,26,27,28,29,30,31,32]. The cfDNA was isolated from stored plasma, using the QIAamp Circulating Nucleic Acid Kit (Qiagen, Hilden, Germany) and quantified on a Qubit fluorometer 1.0 (Invitrogen, Life Technologies, Carlsbad, CA, USA), following the manufacturer’s instructions. The quality test was performed with TapeStation (cfDNA ScreenTape; Agilent Technologies, Santa Clara, CA, USA). The libraries were generated from 13–52 ng of cfDNA and sequenced on a NextSeq 550 system (Illumina, San Diego, CA, USA). The alignment and variant calling was executed through the Agilent SureCall software v.4.2 (Agilent Technologies, Santa Clara, CA, USA), with interpretation and prioritization by Alissa Interpret Analysis Software (Agilent Technologies, Santa Clara, CA, USA).

The specific mutations detected by NGS were confirmed by ddPCR (BioRad, Hercules, USA). The reactions were performed in a 20 µL reaction mix containing 1× droplet PCR supermix, 250 nM of each probe, 450 nM primers and 2–7 µL of cfDNA that was in parallel quantified by ddPCR. The samples were analyzed with the ddPCR BRAF V600 Screening Kit (UniqueAssayIDs dHsaMDV2010027, dHsaMDV2010035, dHsaMDV2010037; BioRad, Hercules, CA, USA) for the BRAF p.V600E/K/R, and the specific assay for p.V600E (UniqueAssayID: dHsaCP2000027, dHsaCP2000028; BioRad, Hercules, CA, USA), according to the manufacturer’s instructions. The droplets were generated and analyzed using the QX200 system (BioRad, Hercules, CA, USA). Positive-, negative- and no template controls were included in each run. The data were analyzed by QuantaSoft analysis software version 1.7.4 (BioRad, Hercules, CA, USA). The samples were defined as positive when three or more FAM-positive droplets were detected with no positive droplets in the negative control. A specific custom assay was designed for tracking the MEK1 p.P124L mutation (UniqueAssayID: dHsaMDS918405961; BioRad, Hercules, CA, USA). The average MAF was calculated from the values collected for each time point by NGS and ddPCR (performed in a total of two or three independent settings). The significance of the different MAFs between the consecutive time points was assessed by Student’s two-tailed *t*-test.

In addition, the BRAF CNV was assessed by ddPCR (UniqueAssayID: dHsaCP2500366; BioRad, Hercules, CA, USA) using as reference the two different probes located on chromosome 14 and 7, (TTC5, UniqueAssayID dHsaCP2506733, and VOPP1, UniqueAssayID: dHsaCP2506684; BioRad, Hercules, CA, USA) [32]. The ddPCR reaction included 1 × ddPCR supermix, primers and probes at a final concentration of 900 nM and 250 nM, respectively, and 2–15 ng of cfDNA in a total volume of 20 μL. To set up the cut-off value for the BRAF gain, 10 healthy volunteer plasma samples (controls) were analyzed. The cut-off was calculated as the CNV mean of controls ± 2SD [33].

### 2.4. Circulating Melanoma Cell Enrichment, Detection and Isolation

The CMCs were enriched from 7.5 ml peripheral blood samples through the CellSearch^®^ system, using the CELLTRACKS^®^ Circulating Melanoma Cell Kit (Menarini Silicon Biosystems, Bologna, Italy) which relies on CD146/HMW-MAA for capture and detection, according to the manufacturer’s instructions. A semi-automated fluorescence-based microscope system, CellTracks Analyzer II (Menarini Silicon Biosystems, Bologna, Italy), was used to identify the circulating melanoma cells. In more detail, an event was classified as a CMC when its morphological features were consistent with those of a cell, and it exhibited the phenotype CD146+, HMW-MAA+, DAPI+ and CD34/45 [34]. The DNA-damaged melanoma cells were identified by the integrated anti-γH2AX antibody which recognizes the phosphorylated form of histone H2AX (γH2AX), correlated to apoptotic chromatin fragmentation [35,36,37,38]. The results were expressed as the total number, and γH2AX-positive CMCs, per 7.5 mL of blood.

The enriched CMCs were isolated by laser capture microdissection (LMD), using the MMI CellCut system (Molecular Machines & Industries GmbH, Eching, Germany) mounted on an ECLIPSE Ti2 microscope (Nikon Corporation, Tokyo, Japan) and subjected to whole-genome amplification (WGA) using the Ampli1™ WGA kit (Menarini, Silicon Biosystems, Bologna, Italy), according to the manufacturer’s instructions with minor modifications. 

## 3. Results

### 3.1. Disease and Treatment Evolution

**Figure 1:**
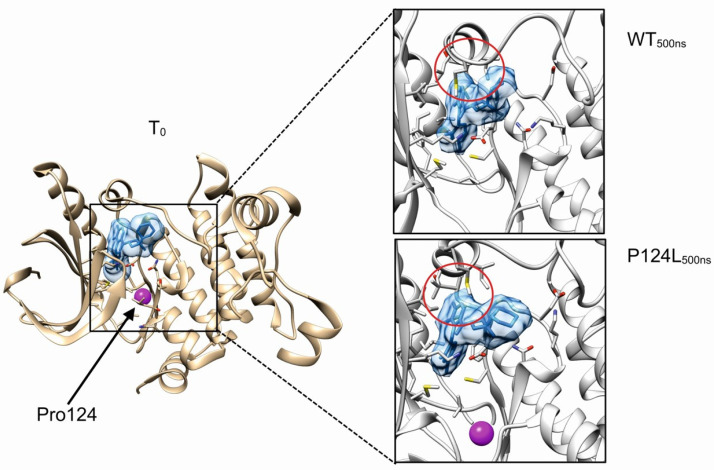
In silico investigation of MEK1 p.P124L mutant. Boxes show the final system states after 500 ns of molecular dynamics simulations. Cobimetinib is represented with solid blue, while purple represents the position of proline 124 residue. Red circle highlights the kinase activation loop. MEK1 residues relevant for the interaction with cobimetinib are represented with sticks.

**Figure 2:**
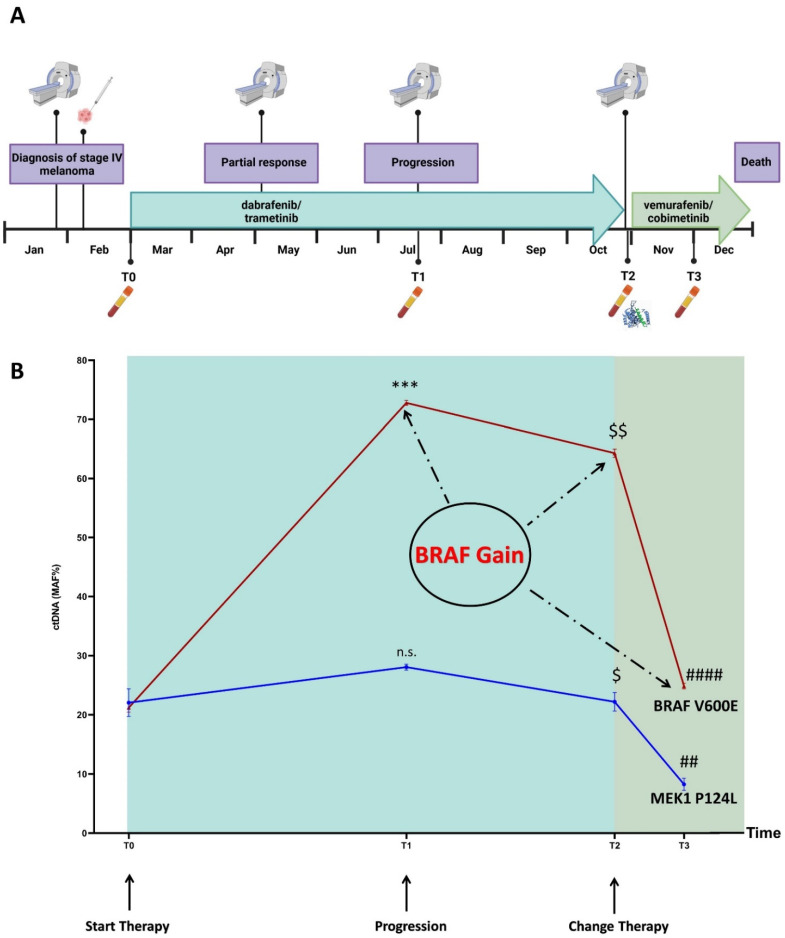
Timeline (**A**) and longitudinal plot (**B**) of BRAF p.V600E and MEK1 p.P124L MAFs (%) detected in ctDNA by NGS and ddPCR (average from values obtained by two or three independent settings). BRAF p.V600E MAFs: 21.01% (T0); 72.75% (T1); 64.30% (T2); 24.86% (T3). MEK1 p.P124L MAFs: 22.05% (T0); 28.05% (T1); 22.20% (T2); 8.26% (T3). $ *p* ≤ 0.05 T2 versus T1; $$ *p* ≤ 0.001 T2 versus T1; ## *p* ≤ 0.001 T3 versus T2; *** *p* ≤ 0.0001 T1 versus T0; #### *p* ≤ 0.000001 T3 versus T2; n.s. = not significant. The timeline was created with BioRender (https://biorender.com/, accessed on 24 June 2022); the plot was performed using GraphPad version 8.0 for Windows (GraphPad Software Inc., San Diego, CA, USA).

A liquid biopsy was used to track the disease evolution of a 42-year-old Caucasian woman diagnosed with metastatic melanoma. Resistance to the dabrafenib and trametinib-targeted therapy resulted in early progression, and based on the results coming from an in silico simulation of the drug–target interaction, an alternative combination of BRAF/MEK inhibitors was suggested (Figure 1 and Figure 2). 

In more detail, in January 2020 the patient presented with spinal pain and underwent magnetic resonance imaging (MRI) and computed tomography (CT) scan, which showed a 3 mm brain metastasis and a diffuse metastatic process involving the lungs, several subcutaneous sites, liver, spleen, bilateral adrenal glands, peritoneal cavity, and the bones at the costal, vertebral, sternal, sacral and pelvic levels. In February 2020, the patient underwent a subcutaneous nodule biopsy that identified a melanoma metastasis harboring the BRAF p.V600E mutation (Figure 2A). The patient received antalgic radiotherapy at the sacral region and brain stereotactic radiosurgery. The combined treatment with dabrafenib/trametinib was started in March 2020. In May 2020, the brain MRI and chest-abdomen CT scan showed a partial response at all of the disease sites; thus, the current therapy was maintained (well tolerated, no side effects). Four months after the beginning of the treatment (July 2020), the brain MRI showed disease progression due to two new metastases, while the chest–abdomen CT showed a maintenance of response at the known disease sites, except for an increase in the bone lesions at pelvis level. The patient received stereotactic radiotherapy for the brain metastases, and radiotherapy for the bone lesions. The combined dabrafenib/trametinib therapy was continued (well tolerated, no side effects). In October 2020, the patient’s clinical condition deteriorated with MRI showing new brain metastases, and the CT scan showed new bone, liver, lung, lymph node, splenic and subcutaneous lesions. In parallel, we performed a ctDNA analysis on both the baseline and progression plasma samples, and detected the MEK1 p.P124L mutation, known to confer resistance to trametinib and/or selumetinib (Figure 2B) [11,39]. Through a molecular dynamics simulation, we assessed that cobimetinib (an alternative MEK inhibitor) would be able to work even in the presence of the p.P124L mutation (Figure 1). In the absence of therapy-related toxicities and since the patient’s general condition was good enough to proceed with the treatment, a switch was made to vemurafenib/cobimetinib targeted treatment, which started in November 2020. As supported by the results obtained from the molecular dynamics simulation, this was likely the best treatment option because the patient was undergoing high-dose steroid therapy due to an epileptic episode, and therefore immunotherapy was not a therapeutic option. Unfortunately, a month and a half later, the patient was hospitalized for deterioration of general clinical conditions, and died a few days later.

### 3.2. Overview

The sequencing of the ctDNA identified the presence, beyond the BRAF p.V600E, of the MEK1 p.P124L mutation and also of the BRAF gain at the progression, conditions that are responsible for the rapid establishment of resistance to the first-line therapy. Since previous studies demonstrated that the MEK1 mutations at codon 124 are able to decrease the interaction between MEK1 and trametinib and/or selumetinib [39,40], we searched for an alternative inhibitor compatible with the presence of this mutation. By molecular dynamics simulation, cobimetinib was predicted to fit the MEK1 binding site, despite the presence of the p.P124L mutation (Figure 1). These findings resulted in a switch to vemurafenib/cobimetinib therapy that induced a rapid decrease in the clone harboring the BRAF p.V600E and MEK1 p.P124L mutations, as tracked via ctDNA analysis (Figure 2B). 

### 3.3. Molecular Dynamics Simulations

The cobimetinib binds MEK1 in a hydrophobic pocket partially overlapping the catalytic site [41]. The proline 124 localizes in a loop connecting the helix αC and the β-strand 4 of MEK1 and its substitution with leucine is predicted to only have a modest impact on the local structure. In particular, the proximal valine 127, a residue involved in the stabilization of cobimetinib, is predicted to maintain the correct orientation useful for stabilizing the inhibitor in the binding pocket. In general, neither the evident local unfolding around the p.P124L mutation site nor the alteration of the kinase activation loop were observed suggesting that this substitution only has a modest or null effect in the binding property of cobimetinib (Figure 1). Evident backbone fluctuations were observed for the loop formed by the MEK1 residues spanning Met219-Ser228. This region is however far more than 10 Å from the mutation site, thus suggesting these movements to be mostly due to an intrinsic flexibility of this loop rather than a long distance effect induced by the mutation. Collectively, our in silico data suggested that cobimetinib is a promising drug candidate for treating patients harboring the MEK1 p.P124L mutation.

### 3.4. Mutation Profiling of ctDNA 

The ctDNA NGS analysis of the samples corresponding to baseline (T0), progression (T1), new line of treatment (T2) and one month after the switch of therapy (T3) revealed a total of six SNVs (Table 1; Table S2, Supplementary Materials) annotated in the Catalogue of Somatic Mutations in Cancer (COSMIC) [42]. Among the SNVs detected at the baseline, two were pathogenic (BRAF p.V600E and MEK1 p.P124L; ACMG classification tool available at VarSome [43]). The progression, that occurred four months later, could have been inferred from the ctDNA trend. Indeed, both the BRAF p.V600E and MEK1 p.P124L mutant allele fractions (MAFs) increased (Figure 2B) and remained high after eight months of dabrafenib/trametinib treatment. 

One month after the therapy shift (T3), we detected a highly significant decrease in the BRAF p.V600E and MEK1 p.P124L MAFs (*p* = 0.0000006 and *p* = 0.0005, respectively). The ddPCR analysis, performed on the same samples, confirmed the NGS results (Table 1).

Although the MAFs of the BRAF p.V600E and MEK1 p.P124L perfectly overlapped at T0, the BRAF p.V600E MAF increased at T1 (time of progression, *p* = 0.00002), T2 and T3 when compared to the MEK1 p.P124L (Figure 2B). We leaned toward a BRAF gain that was confirmed by the ddPCR copy number analysis (CNV), after setting a cut-off for the normal diploid range through the analysis of 10 healthy controls (Table 2). This finding suggests that the overproduction of the BRAF p.V600E may have contributed to decrease the therapeutic inhibitory effect on the MEK-mediated ERK activation even more effectively, because of the preexisting MEK1 p.P124L [40]. Finally, when we considered the absolute amount of ctDNA (copies/ml), instead of the MAF, its trend was different, showing an increase not only, as expected, after progression (T1) and eight months after the beginning of the (ineffective) therapy (T2), but also one month after the new treatment administration (T3) (Table 3). 

### 3.5. Circulating Melanoma Cell Count

**Figure 3:**
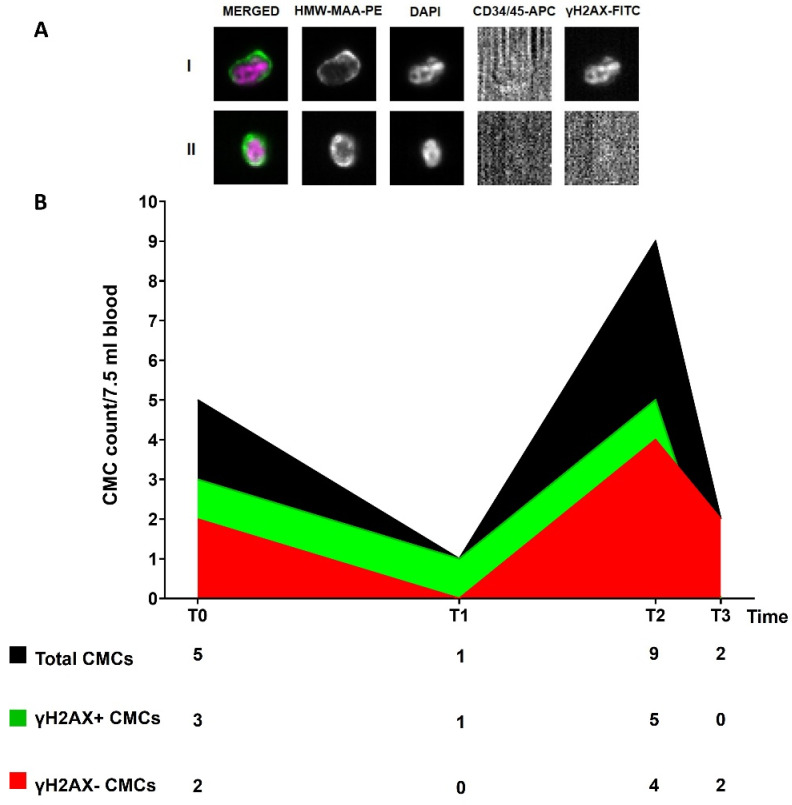
(**A**) Representative images of γH2AX-positive (I) and γH2AX-negative (II) CMCs enriched and detected through the CellSearch^®^ system (10× magnification). Fluorophore-conjugated antibodies were used: anti-High Molecular Weight Melanoma Associated Antigen (HMW-MAA-PE), anti-CD34/45-APC for endothelial cells and leukocytes, respectively, and anti-γH2AX-FITC for DNA-damaged cells. DAPI was used to stain nuclei. No staining is observed in the APC channel in the presence of a CMC; (**B**) Longitudinal plot of CMC count. The table below displays the number of total CMCs and of γH2AX-positive and -negative CMCs at each time point.

We detected the presence of the CMCs at all of the time points (T0-T3). We tried to understand whether the inclusion of a DNA-damage marker (γH2AX) could provide additional information, useful to track disease evolution (Figure 3A). We observed a high CMC number at T0 (5 CMCs), 60% being positive for γH2AX. Then, we observed a decrease at T1 (one CMC γH2AX-positive) when two new brain metastases were observed. At T2, when the MRI and the CT scan revealed the presence of several new lesions, we detected an increase in the CMC number (9 CMCs, 56% γH2AX-positive), as also observed for the ctDNA trend. The CMCs obtained at this time point were laser micro-dissected and their genome was amplified. The ddPCR analysis of the WGA product confirmed the presence of the BRAF p.V600E and MEK1 p.P124L mutations. Finally, we observed a decrease in the CMC count at T3 (one month after the change of therapy) (Figure 3B). All of the CMCs detected at this point were negative for the γH2AX marker. 

## 4. Discussion

In the present study that focused on a challenging clinical case, we exploited an approach that combines a liquid biopsy and in silico-based molecular dynamics’ simulations to identify a successful second-line therapy, and monitor tumor evolution over time in response to the change. Our results can be seen as a follow-up of a previous computational study [40], which demonstrated that activating mutations of MEK1 (one precisely at the amino acidic position 124) can inhibit the effect of trametinib with possibly crucial consequences in the clinic. Moreover, other works have previously shown MEK1 p.P124L to be linked with resistance to trametinib and selumetinib [11,39]. The additional value of our study is that of identifying, by an in silico approach, an alternative drug, among the MEK inhibitors, able to block the target even in the presence of a damaging mutation, and of tracking the response to treatment by liquid biopsy. As a further support of our results, a marked and durable response to cobimetinib was observed in the treatment of a MEK-mutated histiocytic neoplasm, even in the presence of the MEK1 p.P124L mutation [44]. 

These data lead us to think about how the different inhibitors of the same target could have different clinical effects (due mostly to their significantly different biochemical structure), and how, in the case of a hotspot mutation in the target, a combined approach would possibly make the difference. In the era of personalized medicine, where targeted drugs have reached a position of paramount importance, our work points out the necessity of carefully considering that specific hotspots on the target may impair the function of some (but possibly not all) of them. 

Liquid biopsy has emerged as a putative companion diagnostic tool, useful for targeting the tumor heterogeneity, and with a prognostic and/or predictive potential in the presence of a specific cut-off [15,19,20]. Although we reported here a single case that is not sufficient to make a claim for a routinary workflow, this work evidences how a simple and non-invasive approach allows for a strict monitoring of the disease evolution that, when completed in real time, could even help in choosing the best time for shifting to a second-line therapy. The role that in silico tools have gained is also evident, not only in the field of drug design, where their contribution is unquestionable, but also from the perspective of sustaining the choice of specific drugs, even for those that can be considered interchangeable.

From a pragmatic point of view, this was definitely a challenging case with both light and shade, and sometimes difficult to interpret. The tracking of the tumor evolution by the ctDNA trend has offered interesting cues, and confirmed a possible use of ctDNA as a pharmacodynamic marker. Moreover, our results are in line with what was already stated about the potential clinical utility of plasma ctDNA and its ability to capture clonal evolution [45,46,47]. 

Whether observed longitudinally, the BRAF and MEK1 MAFs correlated with the clinical data and imaging examination where available, and were suggestive of progression, resistance and finally of a putative response [47]. Both of the MAFs significantly reduced one month after the change in therapy, as did the number of CMCs, whose count already demonstrated pharmacodynamic and prognostic significance [19]. The main limitation of this study is the absence of blood data from subsequent time points as a means of identifying the dynamics underlying the fast deterioration of the patient’s condition and subsequent death, from a genetic and phenotypic point of view. Therefore, the discussion from this point on may be purely speculative. The major reason leading to the patient’s rapid death after the start of the new regimen, which was administered eight months after the first (ineffective) therapy, probably relies on the highly compromised clinical situation as revealed by imaging and physical examination, as well as on the delay in recommending a change in therapy. In fact, the pilot study that enrolled the patient was not intended to be conducted in real time, and therefore the information about the patient’s intrinsic resistance, already present at baseline, did not become available until several months later.

The CMC counts always remained above the suggested cut-off [19], if we exclude the time of brain metastases progression (probably because of the blood–brain barrier). Even when the cells were stratified according to their DNA-damage characteristics, the count remained above the cut-off, highlighting a compromised situation from the very outset. Indeed, a CMC count ≥ 2 was associated with a significant reduction in OS [19,34], and a CMC count ≥ 1 at baseline with a shorter PFS [48]. Considering that the progression occurred at brain level, it is not surprising that no rise in the CMC count was detected at T1, a predictable consequence already observed [49]. Other works have already run into this problem in the case of brain metastases, suggesting that the blood–brain barrier could also be a hindrance for ctDNA to enter the circulation [45,50,51]. Indeed, a poor amount of ctDNA copies was also detected at T1 in our case. Nevertheless, if we exclude the CMC count at the time of progression that might not be representative of the whole disease for the reasons stated above, the composition of ϒH2AX-positive and -negative CMCs at T0 and T2 were suggestive of a heterogeneous, dynamic disease, as the CMC population was composed roughly by equally damaged and undamaged cells. It is worth noting that the CMCs were 100% undamaged at T3, although fewer when compared to the previous points. This could confirm the aggressiveness of the driving CMC population, as a mirror of a still actively proliferating disease. Despite all of that, and as already emphasized, ours is entirely speculative, having no next time-point sample to determine the evolution of the tumor, and the putative correlation with the DNA-damage status.

Notably, although the MAFs of the BRAF p.V600E and MEK1 p.P124L decreased dramatically, the total amount of the ctDNA increased. This unusual and peculiarly different trend could be explained either as a massive release of cfDNA in response to therapy, or also as a sign of a still highly proliferating disease. Overall, we can hypothesize that the MAF could be representative of the clonal composition of cfDNA (and consequently of the tumor heterogeneity) [52], while the absolute abundance could be considered as a mirror of tumor burden [52,53,54] and proliferation rate. Unfortunately, the lack of any further blood samples does not allow for a precise definition of the role of all of the different “players” (different tumor clones, advanced disease, multiple metastatic sites, compromised conditions) at point T3. Notably, a different trend between the amount of ctDNA (copies/ml) and MAF was already reported, and cautiously interpreted as a sign of response. The authors recommended looking at both the MAF and the total amount of ctDNA for an overall view of the patient’s status [55]. As for our case, again, the availability of a single blood sample collected very close to the therapy switch makes the gathering of definite conclusions very difficult.

Finally, as therapy induces a selection pressure on the tumor, the clone carrying the driver mutation present at the beginning may not be the most informative to be tracked at all of the time points [56]. In this regard, although our NGS panel monitors most of the genes involved in melanoma progression and resistance to treatment (Table S1, Supplementary Materials), we cannot exclude that an epigenetic mutation [57] or one affecting the transcript expression could have guided the tumor proliferation in the final, highly advanced phase. That is currently why the timing of intervention, whether guided by a real-time approach such as liquid biopsy, could be of extreme importance for preventing the tumor outgrowth and uncontrolled proliferation of the newborn resistant clones. The fluctuation of the different SNV MAFs at different time points and the emergence and/or the MAF increase in some of them at T3 (Table 1), are a good example of what was stated above. In our case, since most of the SNVs are classified as variants of uncertain significance (VUS), their role in tumor outgrowth could be a matter of debate, although it would be rather unlikely due to their low MAF at T3. The passenger mutations (those that do not alter fitness but occur in a cell that coincidentally, or subsequently, acquired a driver mutation) might rather be considered as an option for the VUS that we identified [58]. As is already known, the shift of therapy during a response that is assessed not to last long, will increase the chances to respond to the second-line of therapy. Although this is beyond the specific scope of this work, the early identification of intrinsic resistance to targeted therapy could also be important because immunotherapy is indicated in patients with BRAF-mutated melanoma. Therefore, the patients who are unlikely to benefit from BRAFi/MEKi could be treated with checkpoint inhibitors [11].

In conclusion, we believe that a similar approach carried out in real time prior to the rapid escalation of the disease, would have a better chance of success by pinpointing the timing of intrinsic resistance and, concurrently, suggesting a need for a change in the line of therapy. Similar approaches could gain even more importance in the near future when additional targeted drugs will be tested and released.

## 5. Conclusions

This work highlights the importance of detecting mutations responsible for intrinsic resistance before the beginning of the targeted therapy, and the requirement to follow, through liquid biopsy, the response to treatment in order to detect mutations that would confer an acquired resistance. Moreover, it emphasizes the usefulness of defining a combined liquid biopsy/molecular dynamics’ simulation approach, as the monitoring of the treatment response is essential to determine the benefits of new therapies or to avoid the prolonged use of ineffective and potentially toxic treatments.

## Figures and Tables

**Table 1:** ctDNA SNVs detected by NGS at the four time points.

Gene	Position	Coding Change	Amino Acid Change	COSMIC ID	ACMG Classification ^$^	T0 (MAF%)	T1 (MAF%)	T2 (MAF%)	T3 (MAF%)
ALK	2:29,228,936	c.2763C > G	p.F921L	COSM9118654	Uncertain significance	ND	ND	ND	2.5
ATM	11:108,330,374	c.7468C > T	p.L2490F	COSM327924	Uncertain significance	26.1	35.5	27.6	7.8
BRAF *	7:140,753,336	c.1799T > A	p.V600E	COSM476	Pathogenic	20.6	73.1	63.9	24.7
CDKN2A	9:21,971,138	c.221A > C	p.D74A	COSM4163709	Uncertain significance	ND	ND	2.1	ND
HOXD8	2:176,130,574	c.208G > C	p.A70P	COSM3391142	Uncertain Significance	3.8	3.8	4.6	4.7
MEK1 *	15:66,436,825	c.371C > T	p.P124L	COSM1315861	Pathogenic	23.7	27.7	24	9.1

* MAFs detected by ddPCR analysis were: BRAF p.V600E, 21.5% (T0); 72.5% (T1); 63.89% (T2); 24.47% (T3). MEK1 p.P124L, 20.4% (T0); 28.4% (T1); 21.37% (T2); 8.57% (T3). ^$^ ACMG classification tool available at VarSome. Abbreviations: American College of Medical Genetics and Genomics (ACMG); mutant allele fraction (MAF); not detected (ND).

**Table 2:** Summary of BRAF CNV analysis longitudinally monitored.

Time Points	ddPCR Output		Copy Number Assessment
	BRAF/TTC5	BRAF/VOPP1	
T0	2.0	1.9	Diploid
T1	6.5	5.7	Gain
T2	4.8	4.5	Gain
T3	2.7	2.6	Gain

Cut-off: BRAF/TTC5 2.16 ± 0.39 BRAF/VOPP1 2.03 ± 0.56.

**Table 3:** BRAF pV600E and MEK1 p.P124L ctDNA amount (copies/ml plasma) in samples longitudinally collected.

Time Points	BRAF p.V600E (Copies/mL)	MEK1 p.P124L (Copies/mL)
T0	10,432	14,766
T1	8469	1054
T2	11,715	2326
T3	18,080	6578

## Data Availability

The data presented in this study are available from the corresponding author on request.

## Supplementary Materials

The following supporting information can be downloaded at: https://www.mdpi.com/article/10.3390/cancers14174153/s1, Table S1: NGS panel details; Table S2: Characteristics of longitudinal plasma samples and QC metrics of the NGS analysis.
