# Supplementary material for: Investigating the Retained Inhibitory Effect of Cobimetinib against p.P124L Mutated MEK1: A Combined Liquid Biopsy and in Silico Approach
Source: Cancers (Basel). 2022 Aug 27;14(17):4153. doi: 10.3390/cancers14174153 (PMC9454486; doi:10.3390/cancers14174153)
Supplement: Supplementary file 1 [file cancers-14-04153-s001.zip › supplementary table S2.pdf]

Supplementary Table S2. Characteristics of longitudinal plasma samples and QC metrics of the NGS analysis

SAMPLE ID	TOTAL (ng)	MEDIAN DEPTH	1000X targets(%)	500X bases (%)
T0	45.00	3027	97.45	95.91
T1	13.09	1744	96.73	90.49
T2	38.36	3197	97.45	97.65
T3	52.20	2041	96.29	96.54
MEDIAN	41.68	2534	97.09	96.22
